# Signal to noise and b-value analysis for optimal intra-voxel incoherent motion imaging in the brain

**DOI:** 10.1371/journal.pone.0257545

**Published:** 2021-09-23

**Authors:** Harri Merisaari, Christian Federau

**Affiliations:** 1 Department of Diagnostic Radiology, University of Turku, Turku, Finland; 2 Department of Future Technologies, University of Turku, Turku, Finland; 3 Institute for Biomedical Engineering, ETH, Zürich and University Zürich, Zürich, Switzerland; 4 AI Medical, Zürich, Switzerland; University of Queensland, AUSTRALIA

## Abstract

Intravoxel incoherent motion (IVIM) is a method that can provide quantitative information about perfusion in the human body, in vivo, and without contrast agent. Unfortunately, the IVIM perfusion parameter maps are known to be relatively noisy in the brain, in particular for the pseudo-diffusion coefficient, which might hinder its potential broader use in clinical applications. Therefore, we studied the conditions to produce optimal IVIM perfusion images in the brain. IVIM imaging was performed on a 3-Tesla clinical system in four healthy volunteers, with 16 b values 0, 10, 20, 40, 80, 110, 140, 170, 200, 300, 400, 500, 600, 700, 800, 900 s/mm^2^, repeated 20 times. We analyzed the noise characteristics of the trace images as a function of b-value, and the homogeneity of the IVIM parameter maps across number of averages and sub-sets of the acquired b values. We found two peaks of noise of the trace images as function of b value, one due to thermal noise at high b-value, and one due to physiological noise at low b-value. The selection of b value distribution was found to have higher impact on the homogeneity of the IVIM parameter maps than the number of averages. Based on evaluations, we suggest an optimal b value acquisition scheme for a 12 min scan as 0 (7), 20 (4), 140 (19), 300 (9), 500 (19), 700 (1), 800 (4), 900 (1) s/mm^2^.

## 1. Introduction

Intravoxel incoherent motion (IVIM) is a method to separate perfusion effects from thermal diffusion effects from images acquired using diffusion-weighted magnetic resonance [1]. A relatively large amount of experimental evidence consistent with the interpretation that the IVIM method can provide in vivo perfusion information has been collected in the last few years [2]. In particular, the IVIM method has been shown to be applicable in a broad range of brain clinical investigations [3], both in the context of hyperperfused lesions such as in high-grade glioma [4–12], and hypoperfused lesions such as strokes [13–16], vasospasm [17], cerebral lymphoma [18] and cerebral death [19]. In addition, the method has shown promise for the survival prognosis in high-grade brain glioma [20, 21], in differentiating recurrent tumor from radiation necrosis for brain metastases treated with radiosurgery [22], and as a surrogate marker for the progression of cerebral small vessel disease [23, 24]. Unfortunately, IVIM perfusion parameters maps are known to be noisy [25–27], and this is particularly harmful for the detection of hypoperfused lesions, because the quality of the IVIM signal equation fit decreases with decreasing perfusion fraction. Optimizing the acquisition parameters might help reduce this drawback.

Several studies on the effect of b-value distribution on the IVIM reconstruction have been conducted. However, to the best of our knowledge no exhaustive evaluation for the optimal choice of b value in the brain has been conducted, considering the number of averaged repetitions. Lemke et al. studied b value distribution in liver [28]. In the brain, Chabert et al. studied 10 subjects with two b value distributions [29]. Hu et. al [30] studied retrospectively 22 healthy males with 12 b-value sets in low and high groups, suggesting total of eight b-values up to 800–1000 s/mm^2^. A further study in eight healthy subjects with an acquisition with four repetitions in the upper abdomen, an optimization derived from based on Cramér-Rao Lower Bounds suggested to use twice as many b-values as b = 0 images [31, 32]. In [33], simulations and 16 healthy volunteers were analyzed in a rapid measurement setting evaluating two b value distributions. Reproducibility across sites and scanner models with selected b-value set for two of IVIM parameters *f* and *D* parameters only, was evaluated in [34]. Further, inter-rater reliability with eight subjects in various organs, including brain, was studied in [35], and short-term repeatability in [36].

The purpose of this study was to quantify in the brain the conditions to produce optimal IVIM perfusion images. For this, we acquired 20 averages of 16 b values ranging from 0 to 900 s/mm^2^ during a 1-hour scan in four volunteers. We studied the noise characteristics of the trace images in a large number of sub-sets of the acquired b values and number of averages. Finally, we studied and optimized the signal-to-noise properties of the IVIM perfusion maps, using various b set selection strategies.

## 2. Materials and methods

### 2.1 Data acquisition

**Fig 1:**
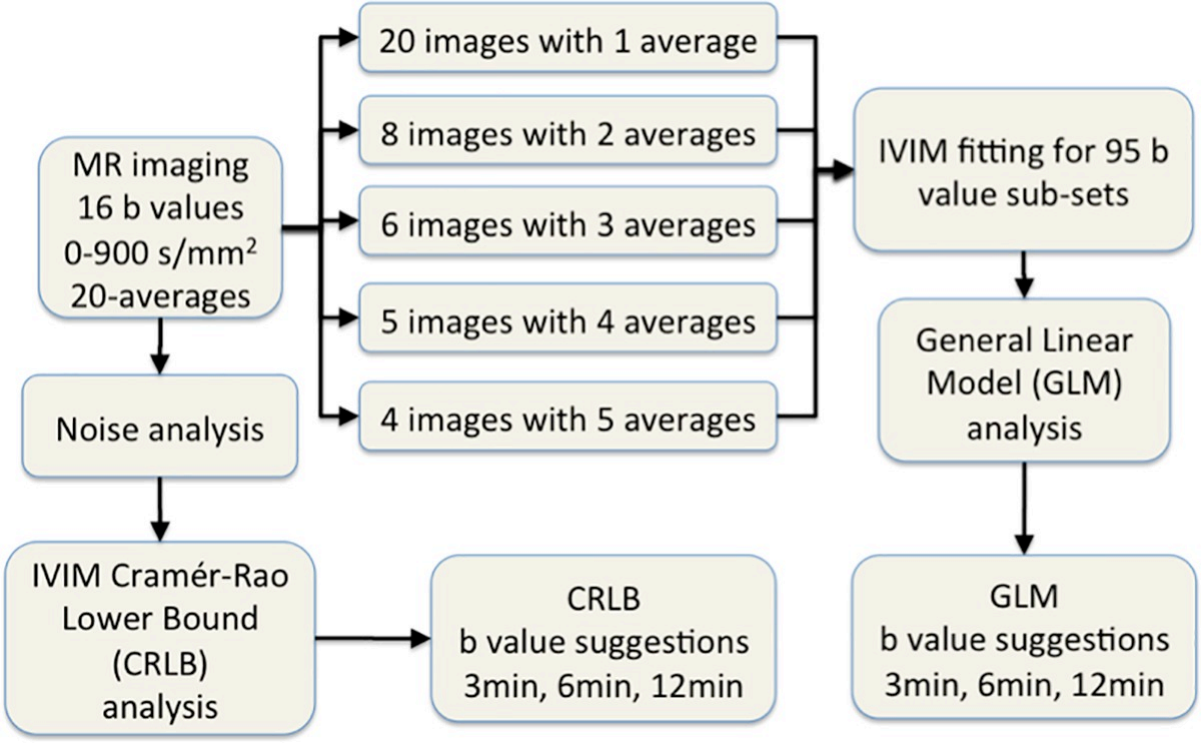
Analysis workflow for optimization of brain IVIM model for number of b values up to 900 s/mm^2^, using 20 repeated scans in four healthy volunteers, with two optimization strategies of General Linear Modelling (GLM) of standard deviation measurements in 95 b value settings, and Cramér-Rao Lower Bound analysis using noise measurements in the same data.

IVIM imaging was performed on a 3-Tesla clinical system (Siemens, Erlangen, Germany) in four healthy volunteers (1 female 25 y, 3 males 26, 38, and 38 y) with parameters: TR/TE 4000/92 ms, FOV 22x22 cm, matrix size 148x148, for a voxel size of 1.5x1.5x6 mm^3^ in approval of local ethics committee. The diffusion weighting was applied in three orthogonal directions from which the trace was computed, with 16 b values 0, 10, 20, 40, 80, 110, 140, 170, 200, 300, 400, 500, 600, 700, 800, 900 s/mm^2^. The acquisition was repeated 20 times. Images with number of averages 1–5 of the measurements were calculated so that in the analysis, there was 20 images including 1 repetition, 10 average images each containing 2 consecutive repetitions, and correspondingly for average images containing 3,4 and 5 consecutive repetitions each. The complete analysis flow is shown in **Fig 1**. This study was approved by the Commission cantonale (VD) d’éthique de la recherche sur I’être humain (Protocole 322/11). Written consent was obtained from all participants.

### 2.2 IVIM model fitting

The IVIM imaging data were post-processed using FSL (FMRIB’s Software Library, www.fmrib.ox.ac.uk/fsl, 5.0.4) [37], and locally written python and C++ code. The b0 images were co-registered to the first b0 image. The co-registered b0 image were averaged, after which all other b-value images were co-registered to the averaged b0 to assess motion during scanning session. The DWI decay curves were fitted with Broyden–Fletcher–Goldfarb–Shanno (BFGS) algorithm implemented in the Dlib-ml C++ library [38]. We followed the fitting procedure described in [26], the fittings were initialized with slopes and perfusion fraction values calculated analytically from the log-transform of the signal and an extrapolated line at b = 0, from the tail values selected according to the available b values (see below) in the images so that all b values > = 300 s/mm^2^ were included. The *D* parameter was fitted first with the values at the tail of the decay curve, and then fixed for the fitting of the *f* and D* parameters. This provided two-stage fittings of the curve with two and three degrees of freedom in the two stages of the fitting procedure, correspondingly. It is to be noted, that in special case of evaluating only three b-values, the fitting procedure is reduced to be identical to determination of initialization of values, as in such situation there are no other data points available for the fitting.

### 2.3 Analysis set-up

Post-processing was done for all acquired images and for three sets of b-values combinations, which all contained the fix 3 b values: b = 0, 200, and 900 s/mm^2^, and which were produced as follow:

- In the first set, b values below 200 s/mm^2^ were selected, following the strategy of maximum sampling in a b value range of the IVIM effect.
- In the second set, b values above 200 s/mm^2^ were selected to follow the approach of maximizing good starting estimate of *D* [39].
- Finally in the third set, one value above 200 s/mm^2^ and one value below 200 s/mm^2^ were selected alternatively, to give emphasis in the mid-range of b-values, as study in [40], has been shown to improve accuracy in estimation of fraction *f* between two exponential decays.

The 3 strategies of b values were then pooled in one ensemble, and further augmented with manually selected b value sets to homogenize the sets and to evaluate previous work on IVIM brain [33], to a final ensemble of 95 different sets of b values (**Appendix A in S1 File**). The selected b values thus roughly represent three b value selection strategies of preferring low b values instead of high ones, preferring high b values instead of low ones, and balancing added b values between low and high b values. They correspond in large part of the b value settings proposed in the literature, within the limits of possible b value sub-sets, which could be generated from the acquired b values. Low b values were defined as the values below 200 s/mm^2^. Values 200, 900 were fixed to follow suggestions of earlier b value set optimization studies [29, 33], and were considered as suggested optimal for optimizing *f* parameter.

### 2.4 Brain segmentation

The brain was segmented in grey matter (GM) and white matter (WM) with average of b0 image in 1 hour acquisition. We applied manually explored b0 image intensity value thresholds for GM and WM, followed by manual edits to remove artifacts (minor intensity inhomogeneity, noise), using ITK-SNAP (www.itksnap.org, version 3.8.0). The voxels of the GM and the WM maps were then pooled for further analysis. The WM region was eroded with one voxel before extracting voxel values, to address potential partial volume effect from neighboring regions.

### 2.5 Standard deviation analysis inside the GM and WM of the original data

To get an overview of the noise characteristics of the original data, the coefficient of variance (i.e. standard deviation divided by the mean) of the voxel intensity values in the GM and WM was calculated for each b value and each average (i.e. 1 to 20 averages) trace images, and plotted as heatmaps for the first of the scanned subjects.

### 2.6 Standard deviation analysis inside the GM and WM of the IVIM parameters

The standard deviation of the IVIM parameters (*f*, *D*, D*) in GM and WM were calculated for the sets of b values and for the averaged images containing 1–5 repetitions. Each set of different b value was analyzed separately for the number of repetitions in average images ranging from 1 to 5. The number of average images were: 20 for 1-average, 10 for 2-average, 6 for 3-average, 5 for 4-average, and 4 for 5-average, total 45 images for each b value setting. The corresponding number of b0 images were 1, 2, 3, 4 and 5. The b0 images were averaged in the latter four partitions.

### 2.7 Optimal b value set and the number of averages for a given scan time of 3 min, 6 min and 12 min

Finally, we assessed the optimal set of b values and number of averages to produce the most homogenous D* maps possible, from all subsets analyzed (see above and **Appendix A in S1 File**). In addition to regression analysis with four subject and 95 different b-value sets, we performed Cramér-Rao Lower Bound (CRLB) search [41] using measured noise in b-values in range 0–900 s/mm^2^ across repetitions, optimizing for all IVIM parameters together and allowing individual b values to have different number of averages to each other.

### 2.8 Statistical analysis

Statistical significance of monotonic upward or downward trends in number of averaged images and number of b values were tested with the Mann-Kendall trend test. Effect of b-value selection, number of repetitions, and the number of b-values were used in multivariate analysis to analyze the relative contribution of these variables to standard deviation IVIM parameter maps in general linear model (GLM) analysis for overall effect of b value set, number of b values in set and number of repetitions to Standard Deviation (SD) (in formula SD ~ b value selection + number of b values + number of repetition). Also, we applied GLM to evaluate effect of including individual b value in b value set. P-values less than 0.05 after Bonferroni correction were considered statistically significant, while raw p-values are reported unless otherwise noted. All statistical tests were done in RStudio environment (v 1.1.383, 2017 RStudio, Inc.).

## 3. Results

### 3.1 Standard deviation inside GM and WM

**Fig 2:**
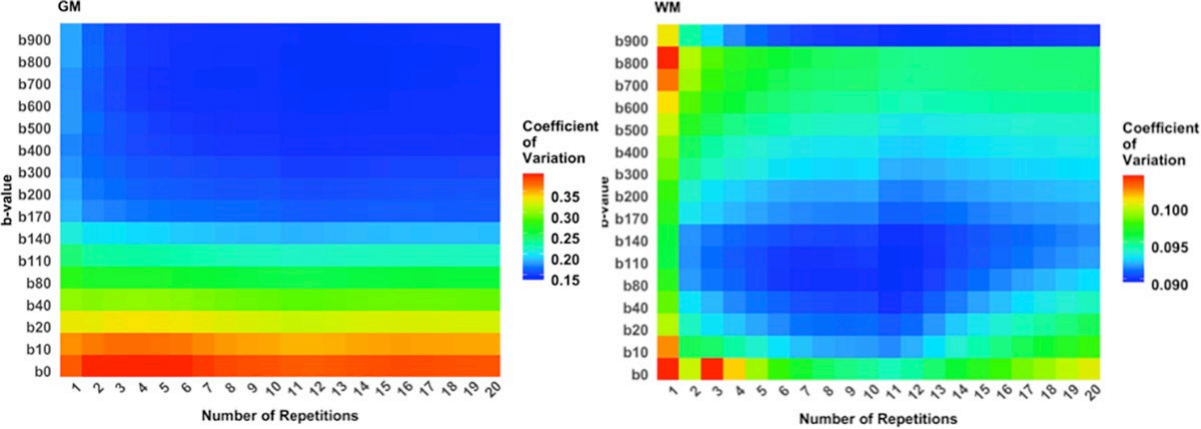
**Coefficient of variation (SD/mean) as a function of b values and number of averages, in the gray matter (left) and the white matter (right) for four healthy volunteers in DWI trace images in b-values ranging from 0 to 900 s/mm**^**2**^**and number of repetitions for signal averaging in 1 to 20.** Gray matter variation begins to drop after b = 170 s/mm^2^ and with 4 repetitions, while white matter variation is generally less and with negligible differences between b-values and repetitions in range 9% to 10.5%, and lowest points around b = 80–140 s/mm^2^ and when using 4 to 13 averages.

The coefficient of variance (CoV) as a function of b value and number of averages in the GM and WM (**Fig 2**) varied in 15%-40% for GM and 9%-10.5% for WM. WM CoV had two peaks at the low number of b-values: One at high b values, due to thermal noise, and interestingly, another one at low b values, most probably due to physiological noise arising from variation in blood flow during the cardiac and respiratory cycles. The peak at low b values was more prominent in the GM compared to the WM. This might probably be due to more prominent capillary network in the GM. We observed that this increase in SD is due to the inclusion of low b-value < 200 s/mm^2^ with a larger signal SD due to physiological noise (see **Fig 2**), and that number of repetitions did not bring notable improvement to it in four evaluated cases and repetitions up to 20.

### 3.2 Effect of number of averages and effect of b-value selection in four subjects

Monotonic trend with Mann-Kendall trend test was statistically significant in the mean WM and GM for all of the three variables *f*, *D*, D* for effect of the number of b-values (p<1.0x10^-6^) and for the number of repetitions (p<0.007), showing that both the number of b-values and the number of repetitions have effect to the IVIM parameter estimates.

When b-value sets having the same number of b-values were averaged and four subjects were combined in the GLM analysis, interaction term between the number of b-values and number of repetitions in WM *D* (p<1.0x10^-6^), and effect of adding variation between subjects to the model in D* and *f* (p<1.0x10^-6^) were found to be significant, and were thus addressed in the subsequent analysis. We observed that the number of b-values was affecting SD of WM and GM in IVIM parameter maps more than the averaging of the signal (p<1.0x10^-6^). Overall, with all b-value sets and number of averages, when testing the effect of an individual b-value set to SD, selection of individual b-value set was explaining SD significantly better than signal averaging (p<1.0x10^-10^).

We also evaluated the b value sets and b values individually to see their effect on SD. The suggested b values which had statistical significance (p<0.001 after Bonferroni correction over b values) in explaining SD, and which made a statistically significant decreasing difference to SD, are listed in **Table 1**. Generally, when other than the fixed b values of 0, 200, 900 s/mm^2^ were considered in all b-value sets, the D* had increased SD when using b values 10, 20, 40, 110, 600 s/mm^2^, in accordance with noise measurements in **Fig 2**. Also, high b-values (with one exception of 10, 110, 500, 600, 700 s/mm^2^) other than 900, increased SD of *f*. The same effect was visible when b values sets with a maximum scan time of 3, 6 and 12 minutes were considered.

**Table 1:** Suggested optimized b value sets for brain IVIM.

	3 min scan	6 min scan	12 min scan
**Gray Matter**			
***f*-optimized**	0 (5), 200 (5), 900 (5) s/mm^2^	0 (10), 200 (10), 900 (10) s/mm^2^	0 (20), 200 (20), 900 (20) s/mm^2^
D*-optimized	0 (4), 200 (4), 800 (4), 900 (4) s/mm^2^	0 (8), 200 (8), 800 (8), 900 (8) s/mm^2^	0 (16), 200 (16), 800 (16), 900 (16) s/mm^2^
***D*-optimized**	0 (2), 10 (2), 20 (2), 40 (2), 80 (2), 140 (2), 200 (2), 900 (2) s/mm^2^	0 (4), 10 (4), 20 (4), 40 (4), 80 (4), 140 (4), 200 (4), 900 (4) s/mm^2^	0 (8), 10 (8), 20 (8), 40 (8), 80 (8), 140 (8), 200 (8), 900 (8)s/mm^2^
CRLB *f*,D*,*D*	0 (2), 20 (3), 110(2), 140 (2), 400 (1), 500 (4), 600 (2) s/mm^2^	0 (2), 20 (5) 140 (6), 400 (9), 500 (1), 600 (5), 700 (1), 800 (1), 900 (2) s/mm^2^	0 (7), 20 (4), 140 (19), 300 (9), 500 (19), 700 (1), 800 (4), 900 (1) s/mm^2^
**White Matter**			
***f*-optimized**	0 (5), 200 (5), 900 (5) s/mm^2^	0 (10), 200 (10), 900 (10) s/mm^2^	0 (20), 200 (20), 900 (20) s/mm^2^
D*-optimized	0 (4), 200 (4), 800 (4), 900 (4) s/mm^2^	0 (8), 200 (8), 800 (8), 900 (8) s/mm^2^	0 (16), 200 (16), 800 (16), 900 (16) s/mm^2^
***D*-optimized**	0 (2), 10 (2), 20 (2), 40 (2), 80 (2), 200 (2), 300 (2), 900 (2) s/mm^2^	0 (4), 10 (4), 20 (4), 80 (4), 200 (4), 300 (4), 900 (4) s/mm^2^	0 (8), 10 (8), 20 (8), 40 (8), 80 (8), 200 (8), 300 (8), 900 (8) s/mm^2^
CRLB *f*,D*,*D*	0 (4), 20 (4), 110 (2), 140 (2), 400 (1), 600 (2), 700 (1) s/mm^2^	0 (5), 20 (9), 40 (1), 80 (1), 110 (9), 140 (1), 170 (2), 500 (3), 600 (1) s/mm^2^	0 (2), 40 (8), 140 (13), 170 (6), 300 (1), 400 (1), 500 (10), 600 (20), 800 (3) s/mm^2^

Suggested optimized b value sets for IVIM in the brain (number of averages in parenthesis), for 3 minutes, 6 minutes and 12 minutes scans for *f*, D*, and *D* parameters using fixed values for *f*, general linear model analysis for D* and *D*, and for all parameters together with Cramér-Rao Lower Bound estimation.

In CRLB analysis where optimized b value settings were aiming for all three IVIM parameters together, the suggested b values were largely in agreement from suggestion from direct SD evaluations with the ensemble of b value sets in GM and in WM. However, the CRLB analysis suggested different number of averages for selected b values, with non-uniform b value averaging and more emphasis towards using small b values with some of the highest available b values.

### 3.3 Optimal b value set and the number of averages for a given scan time of 3, 6 and 12 min

**Fig 3:**
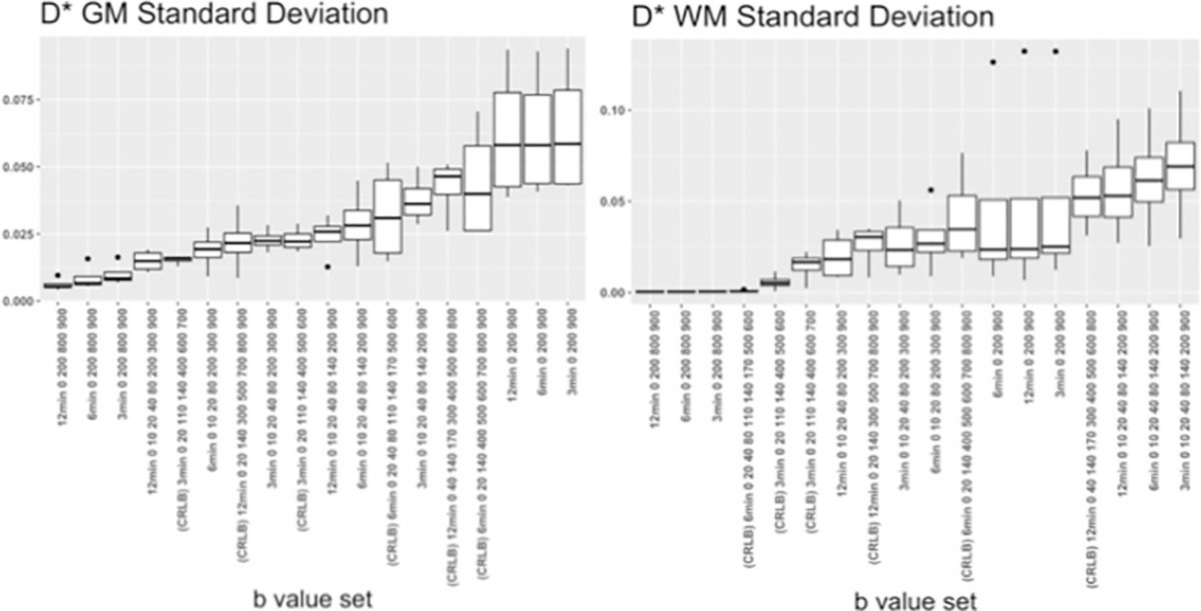
Standard deviations (SD) of IVIM parameter maps D* and *f* in brain grey and white matter over four healthy volunteers, using optimized b value distributions for optimized b value settings from analysis of direct SD measurements, and CRLB using noise estimates, for 3, 6, and 12 minutes scans.

All of the optimized b value sets in **Table 1** were analyzed individually for their SD in GM and WM (D* **Fig 3, S1** and **S2 Figs in S1 File** for *f* and *D*), and mean *f* (**S3 Fig in S1 File**). We found negligible improvement between 3 min to 12 min for b value sub-set of 0, 200, 900 s/mm^2^, which reflects situation where direct analytical estimation of the IVIM parameters is applied from the trace images without fitting. The three b value sub-set was providing reasonably good D* parameter maps in terms of SD, with some expense in quality of *f* values, while noting that low SD for those images may come in expense of losing true D* signal.

In other b value sets, low SD in D* together with reasonable *f* was found with 12 min scan (0 (8), 10 (8), 20 (8), 40 (8), 80 (8), 140 (8), 200 (8), 900 (8) s/mm^2^), and with two b value sub-sets suggested by CRLB for 6 min (0 (5), 20 (9), 40 (1), 80 (1), 110 (9), 140 (1), 170 (2), 500 (3), 600 (1) s/mm^2^), and 12 min (0 (7), 20 (4), 140 (19), 300 (9), 500 (19), 700 (1), 800 (4), 900 (1) s/mm^2^).

**Fig 4:**
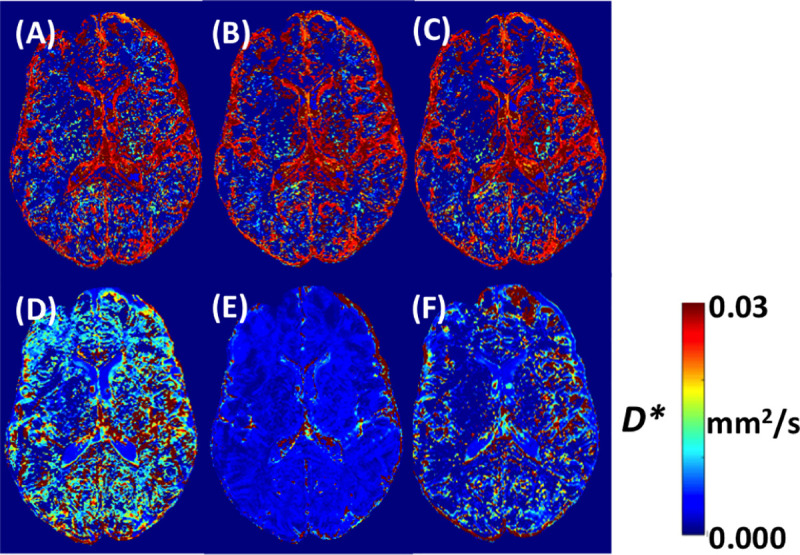
Example of IVIM D* parameter maps with acquisition schemes found best according to their standard deviation for gray and white matter. b values (number of averages) **(A)** 3 min (0 (5), 200 (5), 900 (5) s/mm^2^), **(B)** 6 min (0 (10), 200 (10), 900 (10) s/mm^2^), **(C)** 12 min (0 (20), 200 (20), 900 (20) s/mm^2^), **(D)** 12 min (0 (8), 10 (8), 20 (8), 40 (8), 80 (8), 140 (8), 200 (8), 900 (8) s/mm^2^), **(E)** 6 min (0 (5), 20 (9), 40 (1), 80 (1), 110 (9), 140 (1), 170 (2), 500 (3), 600 (1) s/mm^2^), **(F)** 12 min (0 (7), 20 (4), 140 (19), 300 (9), 500 (19), 700 (1), 800 (4), 900 (1) s/mm^2^).

**Fig 5:**
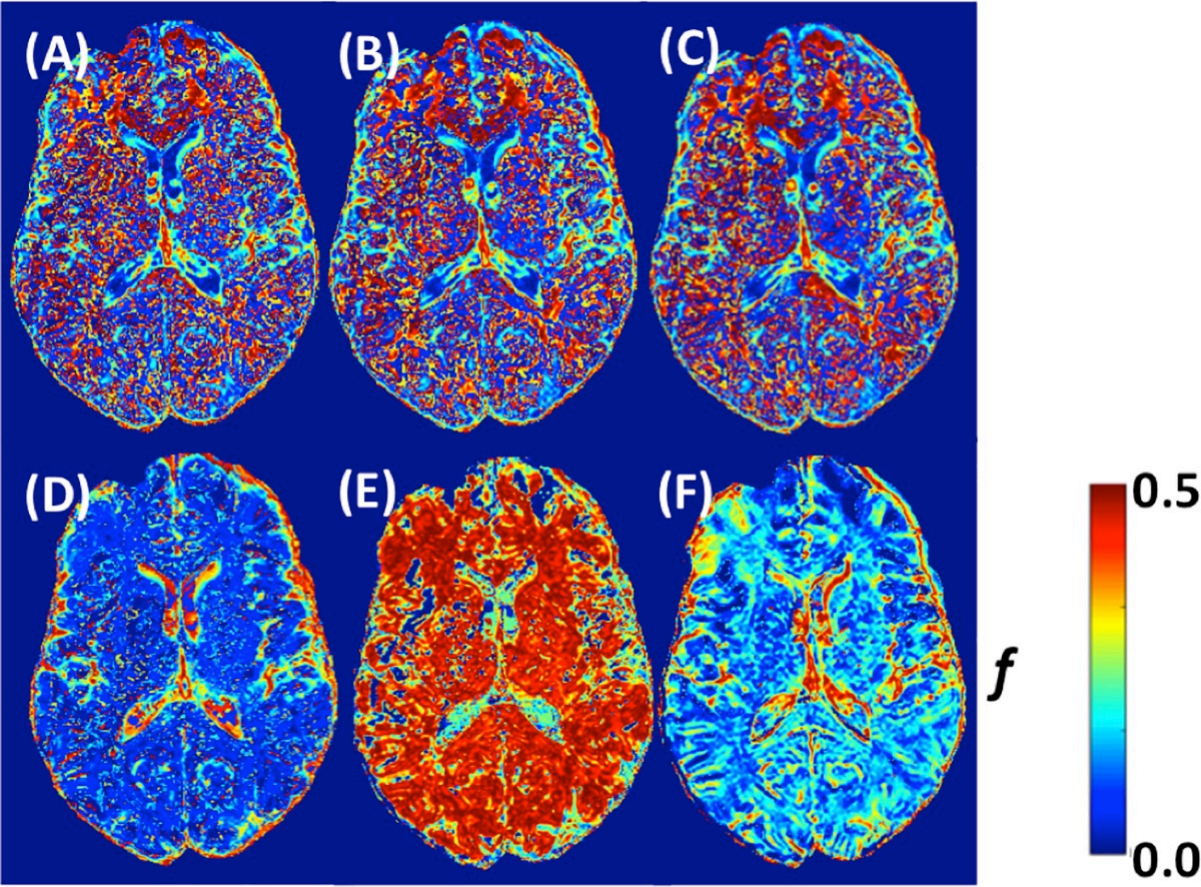
Example of IVIM *f* parameter maps with acquisition schemes found best according to their standard deviation for gray and white matter. b values (number of averages) **(A)** 3 min (0 (5), 200 (5), 900 (5) s/mm^2^), **(B)** 6 min (0 (10), 200 (10), 900 (10) s/mm^2^), **(C)** 12 min (0 (20), 200 (20), 900 (20) s/mm^2^), **(D)** 12 min (0 (8), 10 (8), 20 (8), 40 (8), 80 (8), 140 (8), 200 (8), 900 (8) s/mm^2^), **(E)** 6 min (0 (5), 20 (9), 40 (1), 80 (1), 110 (9), 140 (1), 170 (2), 500 (3), 600 (1) s/mm^2^), **(F)** 12 min (0 (7), 20 (4), 140 (19), 300 (9), 500 (19), 700 (1), 800 (4), 900 (1) s/mm^2^).

In comparison of parameter maps within the same b-value sub-set (0, 200, 900 s/mm^2^), the 3 min, 6 min and 12 min were similar to each other (**A**-**C** in Figs 4 and 5), in D* and *f*. There was apparent difference between optimized 12 min sub-set obtained from GLM approach, and 6 min and 12 min sub-set with CRLB approach (**D**-**F** in Figs **4** and **5**). From all of the evaluated b-value subsets, the 12 min sub-set suggested from CRLB analysis provided most stable parameter maps, for both *f* and D* (**F** in Figs **4** and **5**).

## 4. Discussion

In this extensive study on the dependence of the homogeneity of IVIM parametric maps on the set of values and number of averages in the brain, we found an expected general trend toward an increase in image homogeneity with an increasing number of averages and a less trivial relationship between image homogeneity and the number of different b values used. We found that the inhomogeneity of the IVIM parametric maps increased significantly with the inclusion of b values smaller than 200 s/mm^2^, which showed large SD due to physiological noise, probably mainly due to cardiac pulsation and to a lesser extent to the respiratory cycle. This effect was more pronounced in the GM compared to the WM, but was not observed for the parameter *D*, because the calculation of this parameter does not include values with physiological noise. *D* maps produced with two b values above 200 s/mm^2^ and 2 averaged repetitions each showed already excellent and almost optimal image homogeneity, and only negligible improvements could be obtained if more repetitions or b values were added to the analysis, while *f* and D* benefitted from signal averaging especially when applied to lower b values < 200 s/mm^2^. While there were differences in absolute values *f* and D* between parameter maps using optimized b value sets (Figs **4** and **5**), the IVIM parameter values with 12 min and multiple b values (**D** and **F** in Figs **4** and **5**) were generally within values expected to be found in healthy brain tissue [16]. Overall, taking also into consideration the subjective aspect of the image, the set of parameters 12 min (0 (7), 20 (4), 140 (19), 300 (9), 500 (19), 700 (1), 800 (4), 900 (1) s/mm^2^) seems to provide a good compromise to evaluate f and D* with reasonable low variation.

The choice of optimal scan parameters is in general not trivial, due to the interdependence of a relatively large number of parameters (such as scan time, resolution, TR, TE, bandwidth, for DWI the choice of the profile of the diffusion-sensitizing gradients) and effects (such as hardware related noise, eddy currents, field inhomogeneities, patient related physiological and motion artefacts). In addition, image homogeneity is not necessarily identical with holding maximal physiological or pathological information. Our analysis suggests that the set of b values should be selected with care for IVIM perfusion imaging, particularly when aiming for high-quality D* and *f* parameter maps, and that optimizing for IVIM parameter maps may be required to be performed separately as optimizing for certain parameter comes with the expense of another. The optimal number of repetitions in average images differs between b values, due to need for addressing physiological noise in the low b values, and thermal noise in the high b values. In particular, it seems reasonable to suggest increasing the number of repetitions at low b values (<200s/mm^2^) to average over physiological noise. In addition to compensation of physiological noise, placing more repetitions to mid-range of b values (200–400 s/mm^2^) when making average images improves the estimation of fraction *f*, and D*. We speculate that mid-range b values are located where dominance of D* changes to *D*, and therefore they have an additional contribution in finding the fraction *f* between the two components of the model. A further option to consider to decrease physiological noise effects, although it increases scan time and is difficult to implement in the daily clinical routine because not very practical, is to use triggered acquisitions, such as cardiac gating [42] and respiratory triggering. The use of triggered acquisition has already been shown to decrease measurement variability in IVIM liver imaging [43]. Also, cerebrospinal fluid (CSF), which also undergoes periodic pulsations driven by cardiac and respiratory forces, and participate in the IVIM signal through partial volume [44, 45], could be suppressed using an inversion-recovery pulse [46, 47], or even better, a T2-prepared inversion pulse [48], which permits a better recovery of blood signal with similar suppression of the CSF signal.

There is to our knowledge no study on the choice of b value in the brain for IVIM perfusion imaging. Outside brain, typically 5 to 16 b values have been used to sample perfusion and diffusion IVIM effects [49]. Using Monte-Carlo simulation, Lemke et al. suggested an optimized set of 16 b values for the measurement of a low, medium and high perfusion fractions using Monte-Carlo simulations [28]. Cho et al. optimized the set b value using Monte-Carlo simulations and applied it successfully to IVIM parameters estimation in breast cancer [31]. Pang et al. evaluated different combinations values in prostate cancer [50], Ter Voert et al in the liver [51], and Dyvorne et al. found a subset of 4 optimized b values for the liver [52].

Our study had several limitations. Only four subjects were scanned, while each case was analyzed extensively. Only one sequence of acquisition with maximum b value of 900 s/mm^2^ was used, while sub-sets with the same acquired data was analyzed. Similarly, one acquired fitting approach was used, while the fitting procedure may affect the quality of measured IVIM parameter maps [26]. In our fitting approach, the perfusion fraction and pseudo diffusion were not expected to affect the fit of *D* (with >300 mm/s^2^). We evaluated b value sets only in terms of precision; the acquisition parameter optimization for accuracy or jointly for precision and accuracy was beyond the scope of this work and is left for future investigations. It is left to future studies to assess disease related IVIM parameter values. Preliminary evaluation with one volunteer addressed the effect of the number of repetitions to the distribution of DWI signal intensity values [53]. Neither short term repeatability nor reliability was addressed. Future work should evaluate the repeated measurements with the suggested b-value sets in test-retest setting with larger number of subjects. Our best found b value set requires 12 min scan, which is to be considered relatively high in the clinical setting. Additional work should compare practical usefulness and potential benefit of the created higher quality IVIM parameter maps in specific clinical applications, such as stroke imaging or radiomics feature extraction in tumor imaging.

In conclusion, we evaluated the signal to noise dependence of IVIM trace image data and its effect on the quality of IVIM parameter maps of *D*, *f* and D*. We found that physiological noise at low b value and thermal noise at high b values propagates to the parameter maps. We suggest compensating for this effect by increasing the number of averages in those b values, with additional weighting at mid-range (200–400 s/mm^2^) of b values. Overall, the set of parameters (0 (7), 20 (4), 140 (19), 300 (9), 500 (19), 700 (1), 800 (4), 900 (1) s/mm^2^) seems to provide a good compromise to evaluate f and D* with reasonable low variation.

## Supporting information

S1 File (DOCX)

## References

[1] Le Bihan D, Breton E, Lallemand D, Aubin ML, Vignaud J, Laval-Jeantet M. Separation of diffusion and perfusion in intravoxel incoherent motion MR imaging. Radiology. 1988;168: 497–505. doi: 10.1148/radiology.168.2.3393671 3393671

[2] Federau C. Intravoxel incoherent motion MRI as a means to measure in vivo perfusion: A review of the evidence. NMR Biomed. 2017;30: 1–15. doi: 10.1002/nbm.3780 28885745

[3] Federau C, O’Brien K, Meuli R, Hagmann P, Maeder P. Measuring brain perfusion with intravoxel incoherent motion (IVIM): Initial clinical experience. J Magn Reson Imaging. 2014;39: 624–632. doi: 10.1002/jmri.24195 24068649

[4] Federau C, Meuli R, O’Brien K, Maeder P, Hagmann P. Perfusion Measurement in Brain Gliomas with Intravoxel Incoherent Motion MRI. Am J Neuroradiol. 2014;35: 256–262. doi: 10.3174/ajnr.A3686 23928134 PMC7965752

[5] Bisdas S. Are we ready to image the incoherent molecular motion in our minds? Neuroradiology. 2013;55: 537–540. doi: 10.1007/s00234-013-1192-3 23604821

[6] Togao O, Hiwatashi A, Yamashita K, Kikuchi K, Mizoguchi M, Yoshimoto K, et al. Differentiation of high-grade and low-grade diffuse gliomas by intravoxel incoherent motion MR imaging. Neuro Oncol. 2016;18: 132–141. doi: 10.1093/neuonc/nov147 26243792 PMC4677415

[7] Keil VC, Mädler B, Gielen GH, Pintea B, Hiththetiya K, Gaspranova AR, et al. Intravoxel Incoherent Motion MRI in the Brain: Impact of the Fitting Model on Perfusion Fraction and Lesion Differentiability. Proc J Magn Reson Imaging. 2017;46: 1187–1199. doi: 10.1002/jmri.25615 28152250

[8] Keil VC, Madler B, Schild HH, Hadizadeh DR. Evaluating a Semi-continuous Multi-compartmental Intra-Voxel Incoherent Motion (IVIM) Model in the Brain: How Does the Method Influence the Results in IVIM? In Proc International Society for Magnetic Resonance in Medicine. Toronto, Ontario, Canada; 2015. p. 0348.

[9] Zou T, Rui Q, Yu H, Jiang C, Wang X, Mei Y, et al. Differentiating the histologic grades of gliomas preoperatively using amide proton transfer ‐ weighted (APTW) and intravoxel incoherent motion MRI. NMR Biomed. 2018;31: e3850. doi: 10.1002/nbm.3850 29098732 PMC5757627

[10] Shen N, Zhao L, Jiang J, Jiang R, Su C, Zhang S, et al. Intravoxel Incoherent Motion Diffusion- Weighted Imaging Analysis of Diffusion and Microperfusion in Grading Gliomas and Comparison With Arterial Spin Labeling for Evaluation of Tumor Perfusion. J Magn Reson Imaging. 2016;44: 620–632. doi: 10.1002/jmri.25191 26880230

[11] Wang X, Chen X, Shi L, Dai J. Glioma grading and IDH1 mutational status: assessment by intravoxel incoherent motion MRI. Clin Radiol. 2019;74: 651.e7–651.e14. doi: 10.1016/j.crad.2019.03.020 31014573

[12] Catanese A, Malacario F, Cirillo L, Toni F, Zenesini C, Casolino D, et al. Application of intravoxel incoherent motion (IVIM) magnetic resonance imaging in the evaluation of primitive brain tumours. Neuroradiol J. 2018;31: 4–9. doi: 10.1177/1971400917693025 28643545 PMC5789988

[13] Federau C, Sumer S, Becce F, Maeder P, O’Brien K, Meuli R, et al. Intravoxel incoherent motion perfusion imaging in acute stroke: Initial clinical experience. Neuroradiology. 2014;56: 629–635. doi: 10.1007/s00234-014-1370-y 24838807

[14] Yao Y, Zhang S, Tang X, Zhang S, Shi J, Zhu W, et al. Intravoxel incoherent motion diffusion-weighted imaging in stroke patients: initial clinical experience. Clin Radiol. 2016;71: 938.e11–938.e16. doi: 10.1016/j.crad.2016.04.019 27210244

[15] Suo S, Cao M, Zhu W, Li L, Li J, Shen F, et al. Stroke assessment with intravoxel incoherent motion diffusion-weighted MRI. NMR Biomed. 2016;29: 320–328. doi: 10.1002/nbm.3467 26748572

[16] Federau C, Wintermark M, Christensen S, Mlynash M, Marcellus DG, Zhu G, et al. Collateral blood flow measurement with intravoxel incoherent motion perfusion imaging in hyperacute brain stroke. Neuroradiology. 2019;92. doi: 10.1212/WNL.0000000000007538 31019105

[17] Heit JJ, Wintermark M, Martin BW, Zhu G, Marks MP, Zaharchuk G, et al. Reduced intravoxel incoherent motion microvascular perfusion predicts delayed cerebral ischemia and vasospasm after aneurysm rupture. Stroke. 2018;49: 741–745. doi: 10.1161/STROKEAHA.117.020395 29439196

[18] Yamashita K, Hiwatashi A, Togao O, Kikuchi K, Kitamura Y, Mizoguchi M, et al. Diagnostic utility of intravoxel incoherent motion mr imaging in differentiating primary central nervous system lymphoma from glioblastoma multiforme. J Magn Reson Imaging. 2016;44: 1256–1261. doi: 10.1002/jmri.25261 27093558

[19] Federau C, Nguyen A, Christensen S, Saba L, Wintermark M. Cerebral perfusion measurement in brain death with intravoxel incoherent motion imaging. Neurovascular Imaging. 2016;2: 1–5. doi: 10.1186/s40809-016-0020-7

[20] Puig J, Sánchez-González J, Blasco G, Daunis-I-Estadella P, Federau C, Alberich-Bayarri Á, et al. Intravoxel incoherent motion metrics as potential biomarkers for survival in glioblastoma. PLoS One. 2016;11: 1–14. doi: 10.1371/journal.pone.0158887 27387822 PMC4936699

[21] Federau C, Cerny M, Roux M, Mosimann PJ, Maeder P, Meuli R, et al. IVIM perfusion fraction is prognostic for survival in brain glioma. Clin Neuroradiol. 2017;27: 485–492. doi: 10.1007/s00062-016-0510-7 27116215

[22] Detsky JS, Keith J, Conklin J, Symons S, Myrehaug S, Sahgal A, et al. Differentiating radiation necrosis from tumor progression in brain metastases treated with stereotactic radiotherapy: utility of intravoxel incoherent motion perfusion MRI and correlation with histopathology. J Neurooncol. 2017;134: 433–441. doi: 10.1007/s11060-017-2545-2 28674974

[23] Zhang CE, Wong SM, Uiterwijk R, Staals J, Backes WH, Hoff EI, et al. Intravoxel Incoherent Motion Imaging in Small Vessel Disease: Microstructural Integrity and Microvascular Perfusion Related to Cognition. Stroke. 2017;48: 658–663. doi: 10.1161/STROKEAHA.116.015084 28196940

[24] Wong SM, Zhang CE, van Bussel FCG, Staals J, Jeukens CRLPN, Hofman PAM, et al. Simultaneous investigation of microvasculature and parenchyma in cerebral small vessel disease using intravoxel incoherent motion imaging. NeuroImage Clin. 2017;14: 216–221. doi: 10.1016/j.nicl.2017.01.017 28180080 PMC5288390

[25] Wu WC, Chen YF, Tseng HM, Yang SC, My PC. Caveat of measuring perfusion indexes using intravoxel incoherent motion magnetic resonance imaging in the human brain. Eur Radiol. 2015;25: 2485–2492. doi: 10.1007/s00330-015-3655-x 25693668 PMC4495260

[26] Merisaari H, Movahedi P, Perez IM, Toivonen J, Pesola M, Taimen P, et al. Fitting methods for intravoxel incoherent motion imaging of prostate cancer on region of interest level: Repeatability and gleason score prediction. Magn Reson Med. 2017;77: 1249–1264. doi: 10.1002/mrm.26169 26924552

[27] Ahlgren A, Knutsson L, Wirestam R, Nilsson M, Ståhlberg F, Topgaard D, et al. Quantification of microcirculatory parameters by joint analysis of flow-compensated and non-flow-compensated intravoxel incoherent motion (IVIM) data. NMR Biomed. 2016;29: 640–649. doi: 10.1002/nbm.3505 26952166 PMC5069652

[28] Lemke A, Stieltjes B, Schad LR, Laun FB. Toward an optimal distribution of b values for intravoxel incoherent motion imaging. Magn Reson Imaging. 2011;29: 766–776. doi: 10.1016/j.mri.2011.03.004 21549538

[29] Chabert S, Verdu J, Huerta G, Montalba C, Cox P, Riveros R, et al. Impact of *b*-Value Sampling Scheme on Brain IVIM Parameter Estimation in Healthy Subjects. Magn Reson Med Sci. 2019; 1–11. doi: 10.2463/mrms.mp.2019-0061 31611542 PMC7553810

[30] Hu Y-C, Yan L-F, Han Y, Duan S-J, Sun Q, Li G-F, et al. Can the low and high b-value distribution influence the pseudodiffusion parameter derived from IVIM DWI in normal brain? BMC Med Imaging. 2020;20: 14. doi: 10.1186/s12880-020-0419-0 32041549 PMC7011602

[31] Birkbeck N, Zhang J, Requardt M, Kiefer B, Gall P, Zhou S. Region-specific hierarchical segmentation of MR prostate using discriminative learning. MICCAI Gd Chall prostate MR image segmentation. 2012.

[32] Qinwei Zhang, Yi-Xiang Wang, Ma HT, Jing Yuan. Cramér-Rao bound for Intravoxel Incoherent Motion Diffusion Weighted Imaging fitting. 2013 35th Annual International Conference of the IEEE Engineering in Medicine and Biology Society (EMBC). IEEE; 2013. pp. 511–514. doi: 10.1109/EMBC.2013.6609549 24109736

[33] Meeus EM, Novak J, Dehghani H, Peet AC. Rapid measurement of intravoxel incoherent motion (IVIM) derived perfusion fraction for clinical magnetic resonance imaging. Magn Reson Mater Physics, Biol Med. 2018;31: 269–283. doi: 10.1007/s10334-017-0656-6 29075909 PMC5871652

[34] Grech-Sollars M, Hales PW, Miyazaki K, Raschke F, Rodriguez D, Wilson M, et al. Multi-centre reproducibility of diffusion MRI parameters for clinical sequences in the brain. NMR Biomed. 2015;28: 468–485. doi: 10.1002/nbm.3269 25802212 PMC4403968

[35] Filli L, Wurnig MC, Luechinger R, Eberhardt C, Guggenberger R, Boss A. Whole-body intravoxel incoherent motion imaging. Eur Radiol. 2015;25: 2049–2058. doi: 10.1007/s00330-014-3577-z 25576232

[36] Stieb S, Boss A, Wurnig MC, Özbay PS, Weiss T, Guckenberger M, et al. Non-parametric intravoxel incoherent motion analysis in patients with intracranial lesions: Test-retest reliability and correlation with arterial spin labeling. NeuroImage Clin. 2016;11: 780–788. doi: 10.1016/j.nicl.2016.05.022 27354956 PMC4910187

[37] Jenkinson M, Beckmann CF, Behrens TEJ, Woolrich MW, Smith SM. FSL. Neuroimage. 2012;62: 782–90. doi: 10.1016/j.neuroimage.2011.09.015 21979382

[38] King DE. Dlib-ml: A machine learning toolkit. J Mach Learn Res. 2009;10: 1755–1758.

[39] Rydhög AS, van Osch MJP, Lindgren E, Nilsson M, Lätt J, Ståhlberg F, et al. Intravoxel incoherent motion (IVIM) imaging at different magnetic field strengths: What is feasible? Magn Reson Imaging. 2014;32: 1247–1258. doi: 10.1016/j.mri.2014.07.013 25109587

[40] Merisaari H, Jambor I. Optimization of b-value distribution for four mathematical models of prostate cancer diffusion-weighted imaging using b values up to 2000 s/mm^2^: Simulation and repeatability study. Magn Reson Med. 2015;73: 1954–1969. doi: 10.1002/mrm.25310 25045885

[41] Su J, Rutt B. Optimal Unbiased Steady-State Relaxometry with Phase-Cycled Variable Flip Angle (PCVFA) by Automatic Computation of the Cramér-Rao Lower Bound. In Proc Joint Annual Meeting ISMRM-ESMRMB. Milan, Italy; 2014. p. #3206.

[42] Federau C, Hagmann P, Maeder P, Müller M, Meuli R, Stuber M, et al. Dependence of Brain Intravoxel Incoherent Motion Perfusion Parameters on the Cardiac Cycle. PLoS One. 2013;8: 1–7. doi: 10.1371/journal.pone.0072856 24023649 PMC3758329

[43] Lee Y, Lee SS, Kim N, Kim E, Kim YJ, Yun SC, et al. Intravoxel incoherent motion diffusion-weighted MR imaging of the liver: Effect of triggering methods on regional variability and measurement repeatability of quantitative parameters. Radiology. 2015;274: 405–415. doi: 10.1148/radiol.14140759 25232802

[44] Surer E, Rossi C, Becker AS, Finkenstaedt T, Wurnig MC, Valavanis A, et al. Cardiac-gated intravoxel incoherent motion diffusion-weighted magnetic resonance imaging for the investigation of intracranial cerebrospinal fluid dynamics in the lateral ventricle: a feasibility study. Neuroradiology. 2018;60: 413–419. doi: 10.1007/s00234-018-1995-3 29470603

[45] Becker AS, Boss A, Klarhoefer M, Finkenstaedt T, Wurnig MC, Rossi C. Investigation of the pulsatility of cerebrospinal fluid using cardiac-gated Intravoxel Incoherent Motion imaging. Neuroimage. 2018;169: 126–133. doi: 10.1016/j.neuroimage.2017.12.017 29229579

[46] Wong SM, Backes WH, Zhang CE, Staals J, Van Oostenbrugge RJ, Jeukens CRLPN, et al. On the Reproducibility of Inversion Recovery Intravoxel Incoherent Motion Imaging in Cerebrovascular Disease. Am J Neuroradiol. 2018;39: 226–231. doi: 10.3174/ajnr.A5474 29217741 PMC7410585

[47] Kwong KK, McKinstry RC, Chien D, Crawley AP, Pearlman JD, Rosen BR. CSF-suppressed quantitative single-shot diffusion imaging. Magn Reson Med. 1991;21: 157–163. doi: 10.1002/mrm.1910210120 1943674

[48] Federau C, O’Brien K. Increased brain perfusion contrast with T2-prepared intravoxel incoherent motion (T2prep IVIM) MRI. NMR Biomed. 2015;28: 9–16. doi: 10.1002/nbm.3223 25303668

[49] Taouli B, Beer AJ, Chenevert T, Collins D, Lehman C, Matos C, et al. Diffusion-weighted imaging outside the brain: Consensus statement from an ISMRM-sponsored workshop. J Magn Reson Imaging. 2016;44: 521–540. doi: 10.1002/jmri.25196 26892827 PMC4983499

[50] Pang Y, Turkbey B, Bernardo M, Kruecker J, Kadoury S, Merino MJ, et al. Intravoxel incoherent motion MR imaging for prostate cancer: An evaluation of perfusion fraction and diffusion coefficient derived from different b-value combinations. Magn Reson Med. 2013;69: 553–562. doi: 10.1002/mrm.24277 22488794 PMC3413736

[51] Ter Voert EEGW, Delso G, Porto M, Huellner M, Veit-Haibach P. Intravoxel incoherent motion protocol evaluation and dataquality in normal and malignant liver tissue and comparison to the literature. Invest Radiol. 2016;51: 90–99. doi: 10.1097/RLI.0000000000000207 26405835

[52] Dyvorne H, Jajamovich G, Kakite S, Kuehn B, Taouli B. Intravoxel incoherent motion diffusion imaging of the liver: Optimal b-value subsampling and impact on parameter precision and reproducibility. Eur J Radiol. 2014;83: 2109–2113. doi: 10.1016/j.ejrad.2014.09.003 25277521 PMC4254063

[53] Merisaari H, Federau C. Quantitative Noise Analysis for Increased Homogeneity in Intra-voxel Incoherent Motion (IVIM) Perfusion Imaging in Brain. In Proceedings of the ISMRM 25th Annual Meeting & Exhibition. 2017. p. #3505.

