# Supplementary material for: Signal to noise and b-value analysis for optimal intra-voxel incoherent motion imaging in the brain
Source: PLoS One. 2021 Sep 23;16(9):e0257545. doi: 10.1371/journal.pone.0257545 (PMC8459980; doi:10.1371/journal.pone.0257545)
Supplement: S1 File — (DOCX) [file pone.0257545.s001.docx]

**Supplementary material to manuscript: Signal to Noise and b value Analysis for Optimal Intra-Voxel Incoherent Motion Imaging in the Brain**

Harri Merisaari^1,2^, Christian Federau^3^

^1^ Department of Radiology, University of Turku, Turku, Finland

^2^Department of Future Technologies, University of Turku, Finland

^3^Institute for Biomedical Engineering, ETH, Zürich and University Zürich, Zürich, Switzerland

**Appendix A: Evaluated b value sets**

List of b-values implemented in order to resolve effect of repetitions and b-value sets to standard deviation of resulting IVIM parameter maps.

[0, 10, 20, 40, 80, 110, 140, 170, 200, 300, 400, 500, 600, 700, 800, 900]

[0, 10, 20, 40, 110, 140, 170, 200, 300, 400, 500, 600, 700, 800, 900]

[0, 10, 20, 40, 80, 110, 140, 170, 200, 300, 400, 500, 600, 700, 900]

[0, 10, 20, 40, 80, 140, 170, 200, 300, 400, 500, 600, 700, 800, 900]

[0, 20, 40, 80, 110, 140, 170, 200, 300, 400, 500, 600, 700, 800, 900]

[0, 10, 20, 40, 80, 110, 140, 170, 200, 300, 400, 500, 600, 900]

[0, 10, 20, 40, 80, 110, 140, 170, 200, 300, 500, 600, 700, 900]

[0, 10, 20, 40, 80, 110, 140, 170, 200, 400, 500, 600, 700, 900]

[0, 10, 20, 40, 80, 140, 170, 200, 400, 500, 600, 700, 800, 900]

[0, 20, 40, 110, 140, 170, 200, 300, 400, 500, 600, 700, 800, 900]

[0, 20, 40, 80, 110, 140, 200, 300, 400, 500, 600, 700, 800, 900]

[0, 40, 80, 110, 140, 170, 200, 300, 400, 500, 600, 700, 800, 900]

[0, 10, 20, 40, 80, 110, 140, 170, 200, 300, 400, 500, 900]

[0, 10, 20, 40, 80, 110, 140, 170, 200, 300, 500, 700, 900]

[0, 20, 40, 110, 140, 170, 200, 300, 400, 500, 600, 700, 900]

[0, 20, 40, 80, 140, 170, 200, 400, 500, 600, 700, 800, 900]

[0, 20, 40, 80, 140, 200, 300, 400, 500, 600, 700, 800, 900]

[0, 40, 80, 110, 140, 170, 200, 300, 400, 500, 600, 700, 900]

[0, 80, 110, 140, 170, 200, 300, 400, 500, 600, 700, 800, 900]

[0, 10, 20, 40, 80, 110, 140, 170, 200, 300, 400, 900]

[0, 10, 20, 40, 80, 110, 140, 170, 200, 500, 700, 900]

[0, 110, 140, 170, 200, 300, 400, 500, 600, 700, 800, 900]

[0, 20, 40, 80, 140, 170, 200, 400, 600, 700, 800, 900]

[0, 20, 80, 140, 200, 300, 400, 500, 600, 700, 800, 900]

[0, 40, 110, 140, 170, 200, 300, 400, 500, 600, 700, 900]

[0, 40, 80, 140, 170, 200, 400, 500, 600, 700, 800, 900]

[0, 80, 110, 140, 170, 200, 300, 400, 500, 600, 700, 900]

[0, 10, 20, 40, 80, 110, 140, 170, 200, 300, 900]

[0, 10, 20, 40, 80, 110, 140, 170, 200, 500, 900]

[0, 140, 170, 200, 300, 400, 500, 600, 700, 800, 900]

[0, 20, 80, 200, 300, 400, 500, 600, 700, 800, 900]

[0, 40, 110, 140, 170, 200, 300, 400, 500, 700, 900]

[0, 40, 80, 140, 170, 200, 400, 600, 700, 800, 900]

[0, 80, 110, 140, 170, 200, 300, 400, 500, 600, 900]

[0, 10, 20, 40, 80, 110, 140, 170, 200, 900]

[0, 10, 20, 40, 80, 110, 170, 200, 500, 900]

[0, 10, 20, 40, 80, 140, 170, 200, 500, 900]

[0, 110, 140, 170, 200, 300, 400, 500, 600, 900]

[0, 170, 200, 300, 400, 500, 600, 700, 800, 900]

[0, 40, 110, 140, 200, 300, 400, 500, 700, 900]

[0, 40, 80, 140, 170, 200, 400, 600, 800, 900]

[0, 80, 200, 300, 400, 500, 600, 700, 800, 900]

[0, 10, 20, 40, 80, 110, 140, 200, 900]

[0, 10, 20, 40, 80, 110, 170, 200, 900]

[0, 10, 20, 40, 80, 140, 170, 200, 900]

[0, 110, 140, 170, 200, 300, 400, 500, 900]

[0, 200, 300, 400, 500, 600, 700, 800, 900]

[0, 20, 40, 80, 110, 140, 170, 200, 900]

[0, 40, 110, 140, 200, 300, 500, 700, 900]

[0, 80, 140, 170, 200, 400, 600, 800, 900]0,1

[0, 10, 20, 40, 80, 110, 200, 900]

[0, 10, 20, 40, 80, 140, 200, 900]

[0, 140, 170, 200, 300, 400, 500, 900]

[0, 200, 300, 400, 500, 600, 700, 900]

[0, 200, 400, 500, 600, 700, 800, 900]

[0, 20, 40, 80, 110, 140, 200, 900]

[0, 40, 110, 200, 300, 500, 700, 900]

[0, 40, 80, 140, 170, 200, 400, 900]

[0, 80, 140, 170, 200, 500, 800, 900]

[0, 10, 20, 40, 80, 200, 900]

[0, 140, 170, 200, 300, 400, 900]

[0, 140, 170, 200, 500, 800, 900]

[0, 200, 300, 500, 600, 700, 900]

[0, 200, 500, 600, 700, 800, 900]

[0, 20, 40, 80, 140, 200, 900]

[0, 40, 110, 200, 500, 700, 900]

[0, 40, 140, 170, 200, 400, 900]

[0, 80, 140, 170, 200, 400, 900]

[0, 10, 20, 40, 200, 900]

[0, 110, 200, 500, 700, 900]

[0, 140, 170, 200, 600, 900]

[0, 170, 200, 300, 400, 900]

[0, 200, 300, 500, 700, 900]

[0, 200, 600, 700, 800, 900]

[0, 20, 80, 140, 200, 900]

[0, 40, 140, 170, 200, 900]

[0, 80, 140, 170, 200, 900]

[0, 10, 20, 200, 900]

[0, 110, 200, 500, 900]

[0, 140, 170, 200, 900]

[0, 170, 200, 300, 900]

[0, 170, 200, 600, 900]

[0, 200, 500, 700, 900]

[0, 200, 700, 800, 900]

[0, 20, 80, 200, 900]

[0, 80, 170, 200, 900]

[0, 10, 200, 900]

[0, 110, 200, 900]

[0, 140, 200, 900]

[0, 170, 200, 900]

[0, 200, 300, 900]

[0, 200, 500, 900]

[0, 200, 800, 900]

[0, 80, 200, 900]

[0, 200, 900]

**
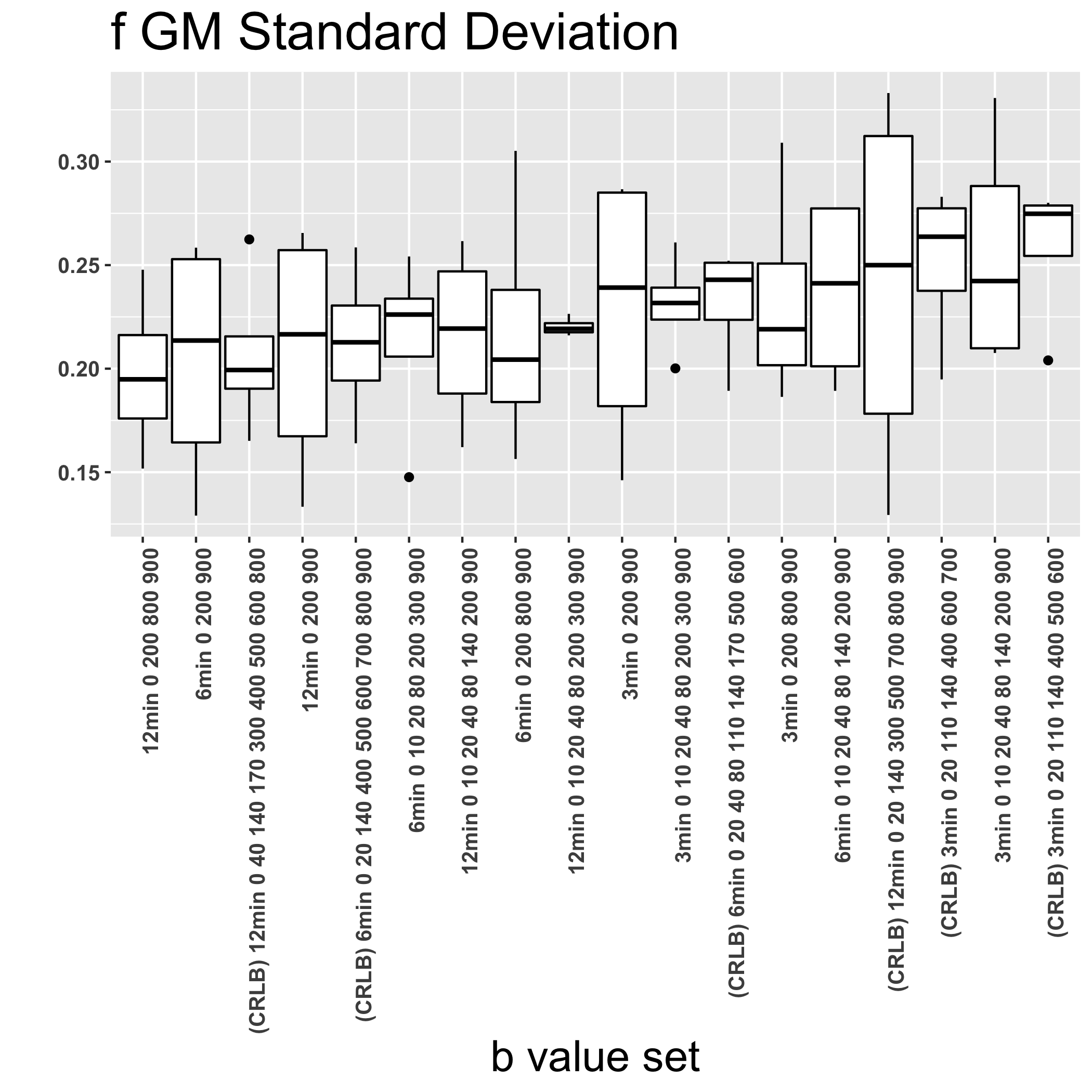

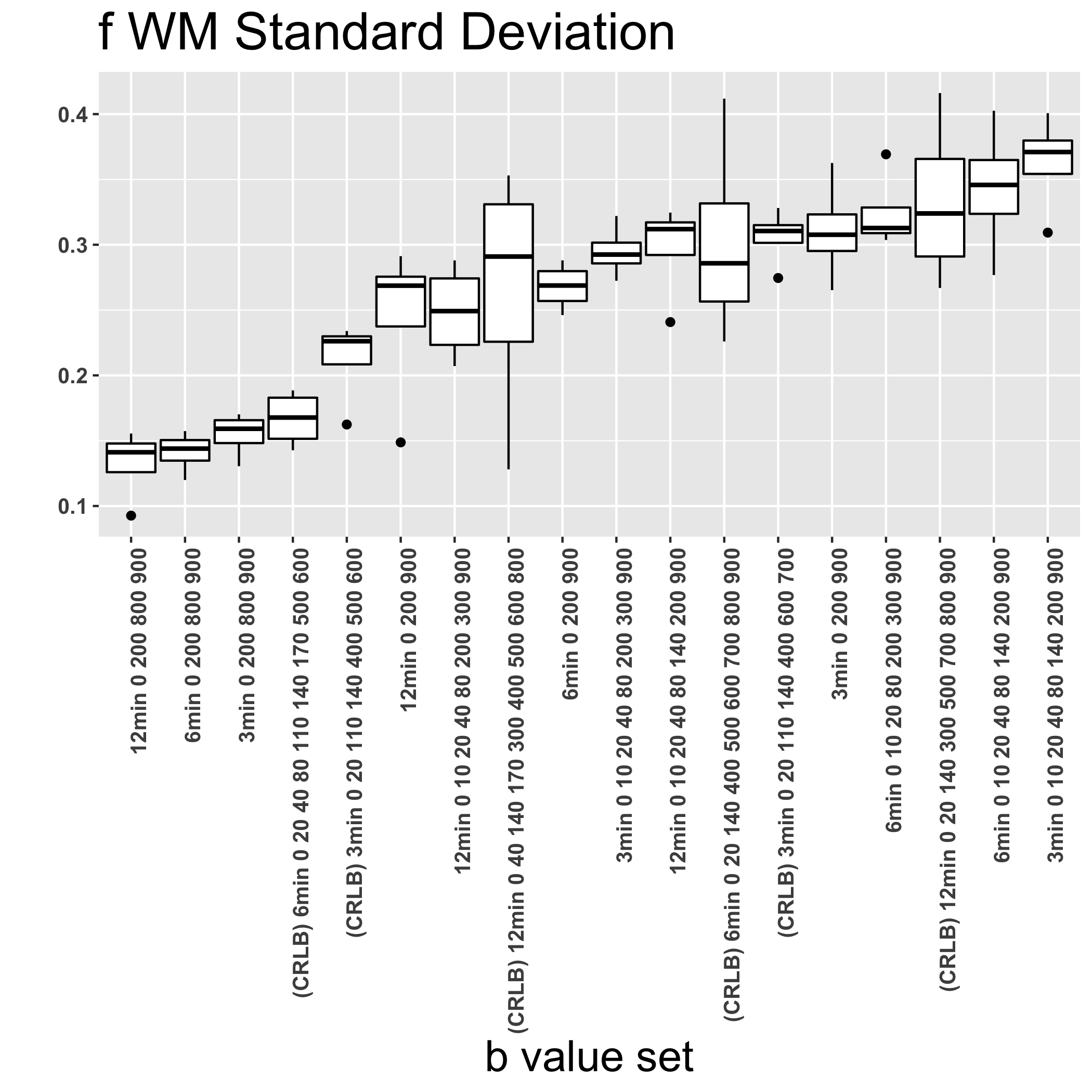
**

**Supplementary Figure S1**: Standard deviations (SD) of IVIM *f* parameter maps in brain gray and white matter over four healthy volunteers, using optimized b value distributions for optimized b value settings from analysis of direct SD measurements, and Cramér-Rao Lower Bound (CRLB) analysis using noise estimates, for 3, 6, and 12 minutes scans.


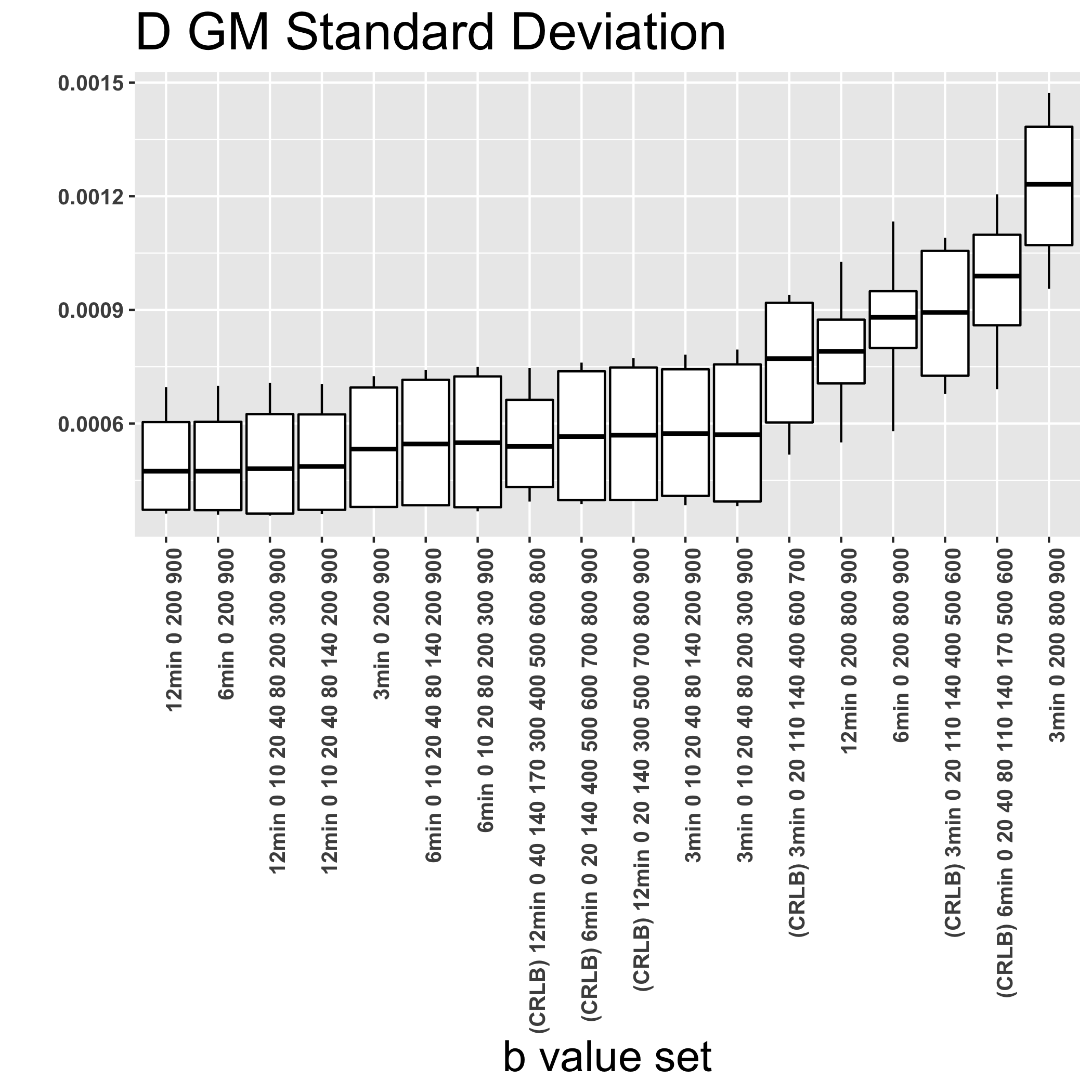

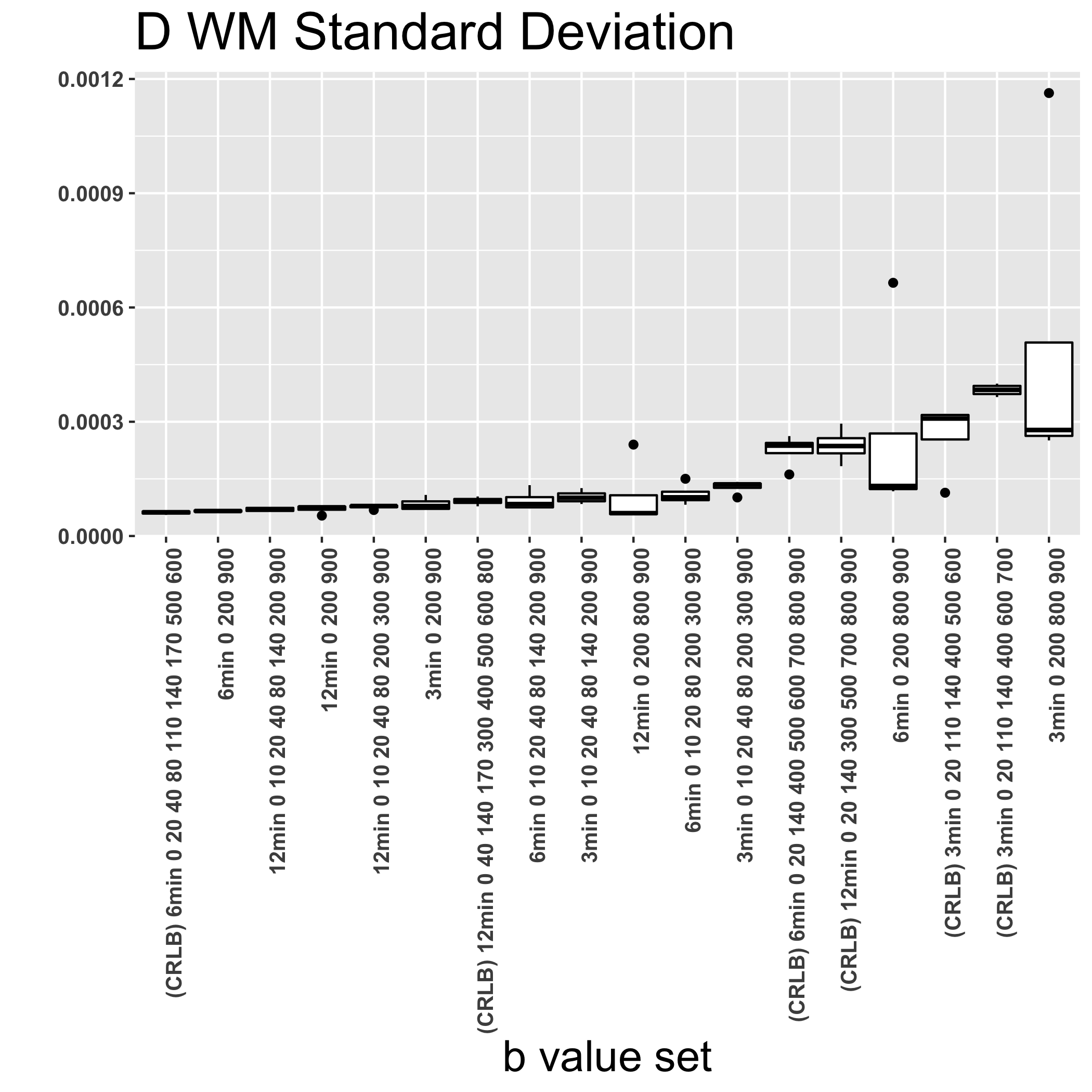


**Supplementary Figure S2**: Standard deviations (SD) of IVIM *D* parameter maps in brain gray and white matter over four healthy volunteers, using optimized b value distributions for optimized b value settings from analysis of direct SD measurements, and Cramér-Rao Lower Bound (CRLB) analysis using noise estimates, for 3, 6, and 12 minutes scans.

**
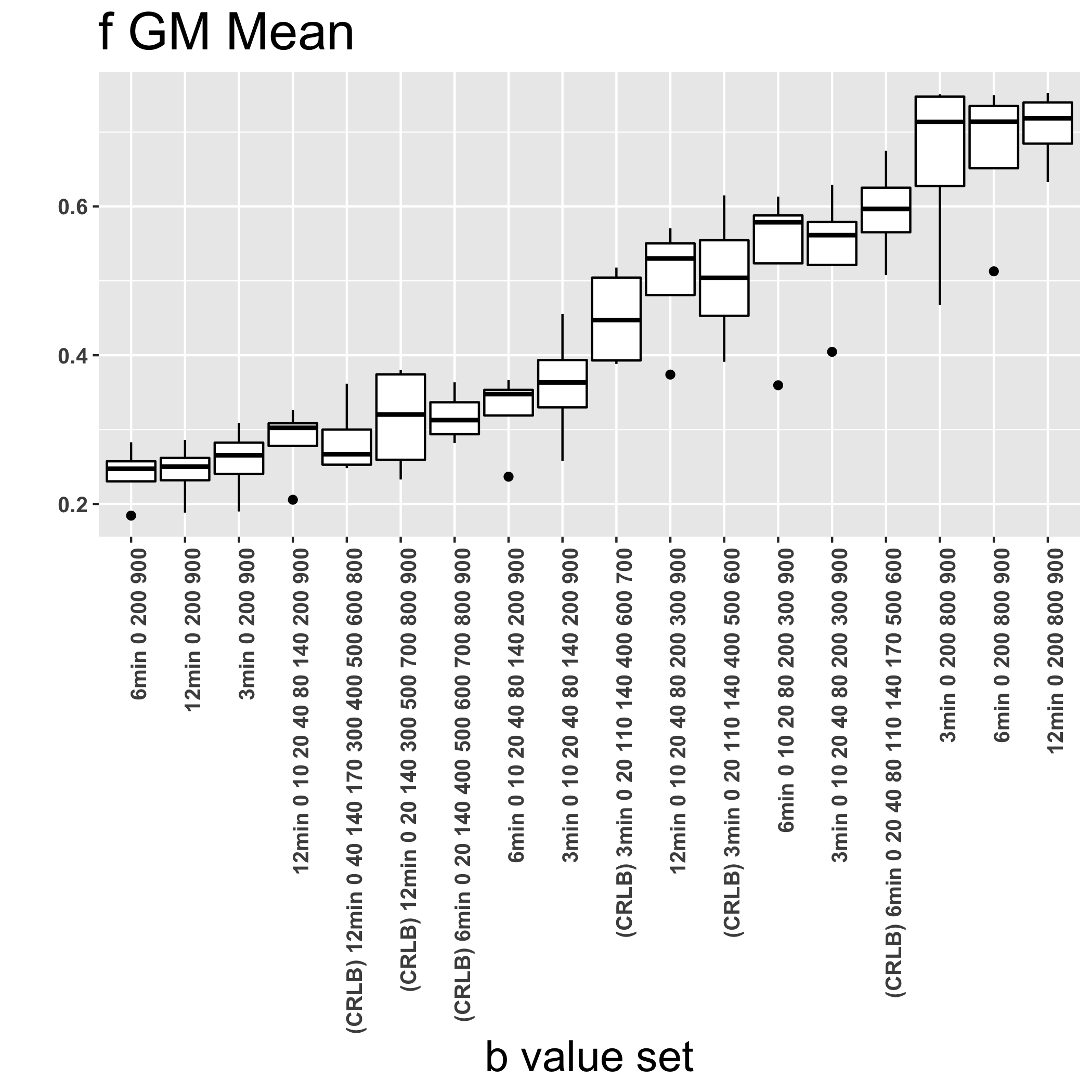

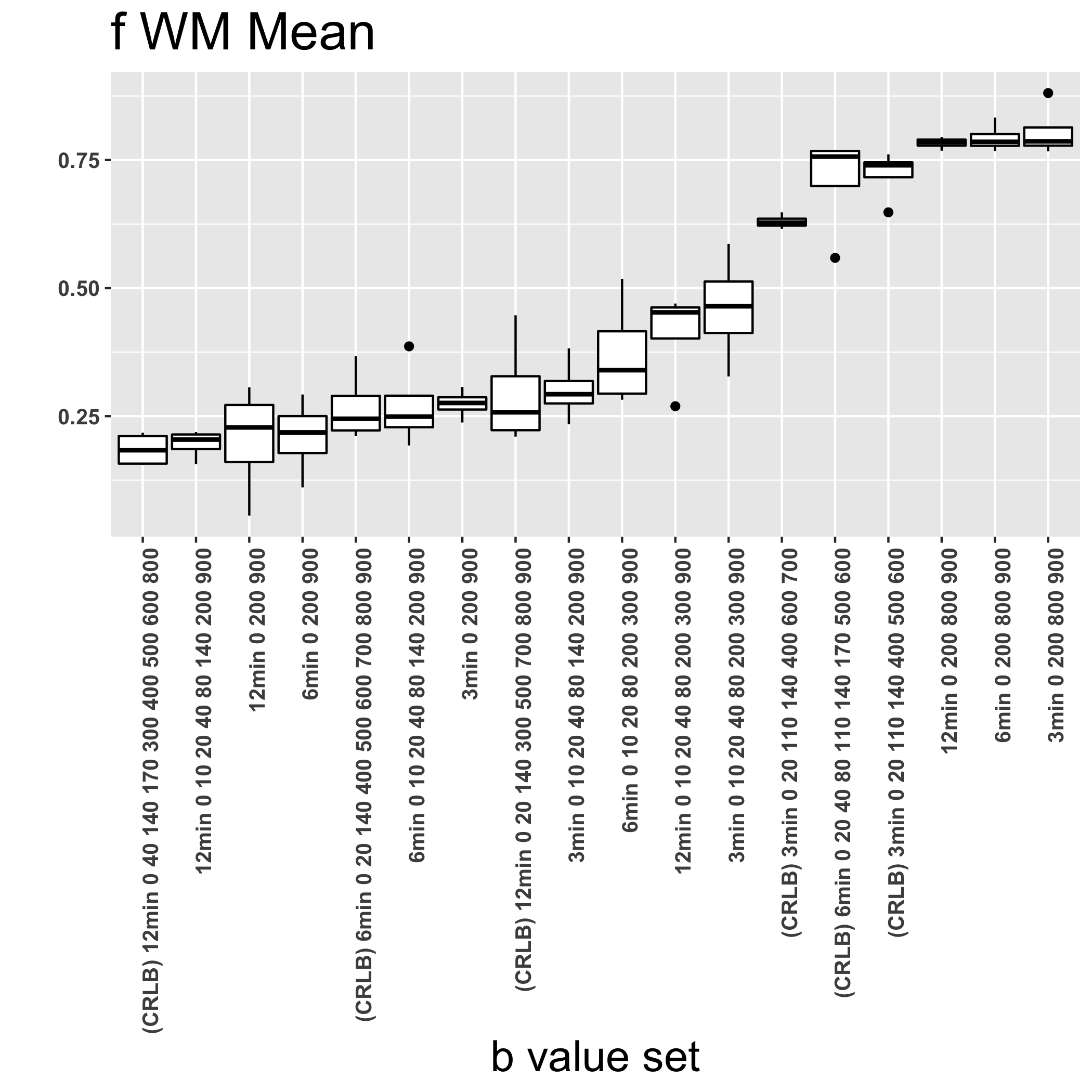
**

**Supplementary Figure S3**: Mean in tensity values of IVIM *f* parameter maps in brain gray and white matter over four healthy volunteers, using optimized b value distributions for optimized b value settings from analysis of direct SD measurements, and Cramér-Rao Lower Bound (CRLB) analysis using noise estimates, for 3, 6, and 12 minutes scans. b value sub-sets having *f* close to 0.5 or above are to be considered having excessinve amount of fraction in the faster diffusion component *D** of IVIM model, and corresponding b value sets may not be measuring *D** properly.
